# Value of 3D-TOF MR angiography and 4D-dynamic contrast-enhanced MRI in the assessment of spontaneous posterior cavernous sinus dural arteriovenous fistula

**DOI:** 10.1590/0004-282X-ANP-2020-0341

**Published:** 2021-05-30

**Authors:** Francisco Bermal CAPARROZ, Lucas Giansante ABUD, Rafael Gouveia Gomes de OLIVEIRA, Daniel Giansante ABUD, Soraia Ramos Cabete FABIO

**Affiliations:** 1 Documenta, Hospital São Francisco, Departamento de Radiologia e Diagnóstico por Imagem, Ribeirão Preto SP, Brazil. Documenta Hospital São Francisco Departamento de Radiologia e Diagnóstico por Imagem Ribeirão Preto SP Brazil; 2 MED Medicina Diagnóstica, Hospital São Lucas, Departamento de Radiologia e Diagnóstico por Imagem, Ribeirão Preto SP, Brazil. MED Medicina Diagnóstica Hospital São Lucas Departamento de Radiologia e Diagnóstico por Imagem Ribeirão Preto SP Brazil; 3 Universidade de São Paulo, Faculdade de Medicina de Ribeirão Preto, Divisão de Neurorradiologia Intervencionista, Ribeirão Preto SP, Brazil. Universidade de São Paulo Universidade de São Paulo Faculdade de Medicina de Ribeirão Preto Divisão de Neurorradiologia Intervencionista Ribeirão Preto SP Brazil; 4 Hospital da Unimed de Ribeirão Preto, Departamento de Neurologia, Ribeirão Preto SP, Brazil. Hospital da Unimed de Ribeirão Preto Departamento de Neurologia Ribeirão Preto SP Brazil

**Figure 1.**
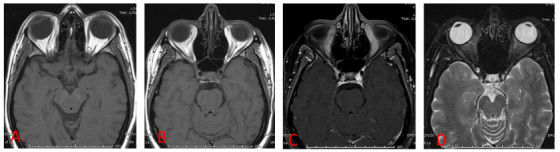
T1-weighted spin-eco (A and B), T2-weighted fat-saturated (A), and post-contrast T1-weighted (B) images from the orbital MR imaging showed no abnormalities. Of note, there are no signs of ocular proptosis or superior ophthalmic vein dilatation.

**Figure 2.**
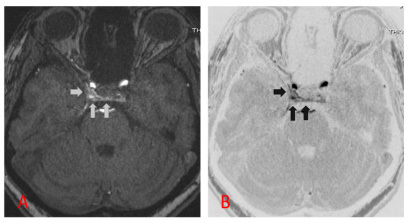
3D time-of-flight MR angiography (A) and the inverted window of this sequence (B) showed a subtle enlargement of the right cavernous sinus and parasellar high signals (arrowhead).

**Figure 3.**
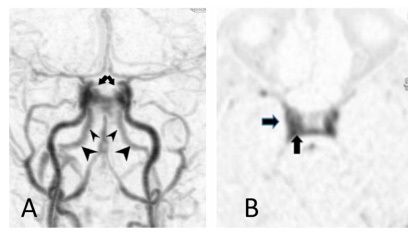
4D-dynamic contrast-enhanced MR angiography in early arterial phase (A) disclosed a slight asymmetric enhancement of bilateral cavernous sinus (arrows), mainly on the right (arrows), in the subsequent phases (B). This cavernous sinus dural arteriovenous fistula is draining downward through the inferior petrous sinus (A: arrowhead) and not upward through the superior ophthalmic vein, which is present in 9-12% of all cavernous sinus dural arteriovenous fistulas.

**Figure 4.**
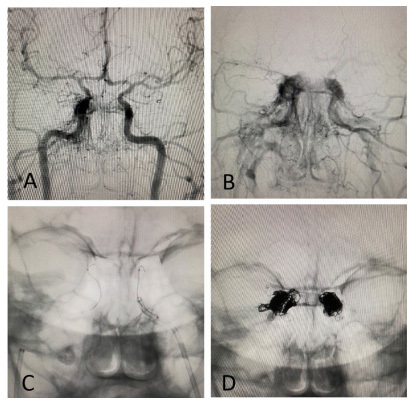
Digital subtraction angiography revealed a bilateral slow-flow posterior cavernous sinus dural arteriovenous fistula, mainly on the right (A and B). Cavernous sinus dural arteriovenous fistula was successfully treated by venous approach (C). Post-embolization of the cavernous sinus was performed through the inferior petrous sinus, using coils and liquid embolic agents (Phil®️) (D).

A 46-year-old female patient presented symptoms of complete right oculomotor nerve palsy without proptosis. Orbital magnetic resonance (MR) imaging showed no abnormalities (Figure1). 3D time-of-flight MR angiography revealed high signal intensity in the right cavernous sinus (Figure 2). This isolated finding has a 10-15% rate of false-positive in the diagnosis of dural arteriovenous fistula (DAVF)^1^. Additional 4D-dynamic contrast-enhanced MR angiography evidenced an early asymmetric enhancement of bilateral cavernous sinus, mainly on the right, draining downward through the inferior petrous sinus (Figure 3)^2^^,^^3^^,^^4^^,^^5^. Digital subtraction angiography confirmed a posterior cavernous sinus DAVF, and endovascular treatment was prescribed (Figure 4).
